# Fractional Generalizations of Maxwell and Kelvin-Voigt Models for Biopolymer Characterization

**DOI:** 10.1371/journal.pone.0143090

**Published:** 2015-11-24

**Authors:** Bertrand Jóźwiak, Magdalena Orczykowska, Marek Dziubiński

**Affiliations:** Faculty of Process and Environmental Engineering, Lodz University of Technology, Łódź, Poland; Northwestern Polytechnical University, CHINA

## Abstract

The paper proposes a fractional generalization of the Maxwell and Kelvin-Voigt rheological models for a description of dynamic behavior of biopolymer materials. It was found that the rheological models of Maxwell-type do not work in the case of modeling of viscoelastic solids, and the model which significantly better describes the nature of changes in rheological properties of such media is the modified fractional Kelvin-Voigt model with two built-in springpots (MFKVM2). The proposed model was used to describe the experimental data from the oscillatory and creep tests of 3% (w/v) kuzu starch pastes, and to determine the values of their rheological parameters as a function of pasting time. These parameters provide a lot of additional information about structure and viscoelastic properties of the medium in comparison to the classical analysis of dynamic curves *G’* and *G”* and shear creep compliance *J(t)*. It allowed for a comprehensive description of a wide range of properties of kuzu starch pastes, depending on the conditions of pasting process.

## Introduction

Biopolymers produced by living organisms can be divided into three main groups [1]: polysaccharides (cellulose, starch, pectin, chitin, glycogen, inulin), polypeptides (proteins) and nucleic acids (DNA and RNA). Biopolymers are characterized by significant sensitivity to physical and chemical factors, therefore, careful examination of their structure and rheological properties requires the use of non-invasive measurement methods. One of them is the oscillation technique. It consists in subjecting the material sample to sinusoidal strain or stress and recording its reaction. This measurement does not affect the structure of the medium—as long as the amplitude is not too large—and can be used to control the processes taking place in time [2].

The obtained experimental data from dynamic tests in the form of storage modulus *G’* (responsible for elastic properties of the material) and loss modulus *G”* (representing viscous characteristics of medium) can be described by rheological models. The classical phenomenological rheological models are composed of two types of elements—spring and dashpot. The spring element behaves in accordance with Hooke’s law and represents ideal elastic response of a material to applied stress; while the viscous element obeys the law of Newton and describes the energy losses due to viscous dissipation [3–5]. Depending on the number and manner of combining of these basic elements, in the literature there have been proposed a number rheological models [3,6–8]. However, their practical use is limited to media with not very complex rheological properties [9–11], which are certainly not biopolymers. Much more possibilities in the description of the behavior of such materials provide fractional rheological models, created on the basis of differential calculus of fractional order 0 ≤ *α* ≤ 1 [12]. Fractional model in terms of construction differs from a classical model in that the certain standard components as a spring or dashpot are replaced by Scott-Blair elements (springpots). Each springpot can be understood as a component having intermediate properties between a purely elastic element (for which *α* = 0) and a perfectly viscous element (for which *α* = 1) [13,14].

Fractional rheological models allow to describe the dynamic behavior of a medium with a single constitutive equation which contains a certain number of parameters that are the constants determining viscoelastic properties of a given material [15]. The identification of these values is so-called reverse problem—in the first place, approximation of the experimental data with trigonometric functions is made, and then rheological parameters of the applied model are determined. Obtained quantities allow for a comprehensive assessment of the medium structure [16–19].

In the literature, there are two basic phenomenological models that describe the rheological behaviors of viscoelastic materials. These are: Maxwell model and Kelvin-Voigt model [3,20,21]. This division results from the way of connecting the elastic Hooke and viscous Newton elements (serial or parallel). Two-element models take into account only a single relaxation time and a single elastic modulus, which practically excludes the possibility of their use for the characterization of viscoelastic media in a wide range of oscillation frequencies [6,22,23].

The aim of the study was to propose and compare the new modified forms of Maxwell and Kelvin-Voig fractional rheological models to improve the description of the dynamic behavior of biopolymeric materials in the area of viscoelastic plateau and within the scope of the smallest oscillation frequencies *ω*. Finding the optimal rheological model whose parameters are simultaneously the material constants, allows for a comprehensive assessment of the structure and viscoelastic properties of the medium.

## Materials and Methods

### Kuzu starch pastes

**Fig 1:**
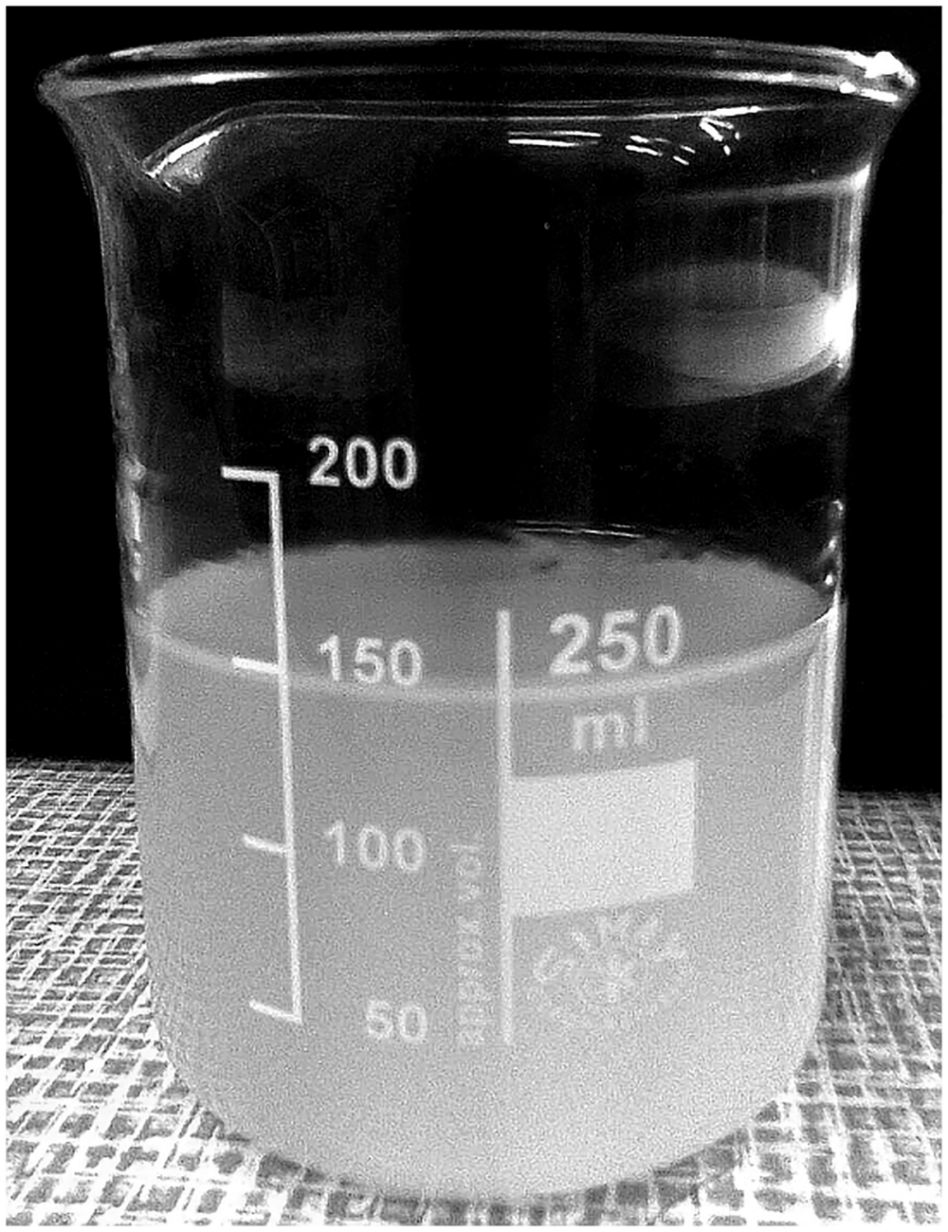
Sample of 3% (w/v) kuzu starch paste for oscillatory and creep tests.

The rheological studies involved 3% (w/v) Japanese kuzu starch pastes (Terrasana, Netherlands). Pasting process of aqueous starch suspensions was carried out at 90°C for 15, 30, 45, 60 or 75 min, while stirring with a magnetic stirrer at a constant rotation speed of 300 rpm. After 24 hours, the obtained pastes (Fig 1) were subjected to oscillatory and creep tests by means of rotary rheometer Physica MCR 301 (Anton Paar, Austria) with a cone-plate configuration (cone diameter– 60 mm, cone slope– 1°, gap width– 117 μm). Rheological measurements conducted at a constant temperature of 25°C included the determination of storage modulus *G'* and loss modulus *G”* for oscillation frequencies *ω* in the range of 6.3·10^−4^ to 450 s^-1^, and at a given sinusoidal strain with 3% amplitude, within the linear viscoelastic region. In addition, the 2500-second measurements of shear creep compliance *J(t)* at a specified constant shear stress value of 1 Pa, also within the range of linear viscoelasticity, were performed.

### Maxwell-type models

**Fig 2:**
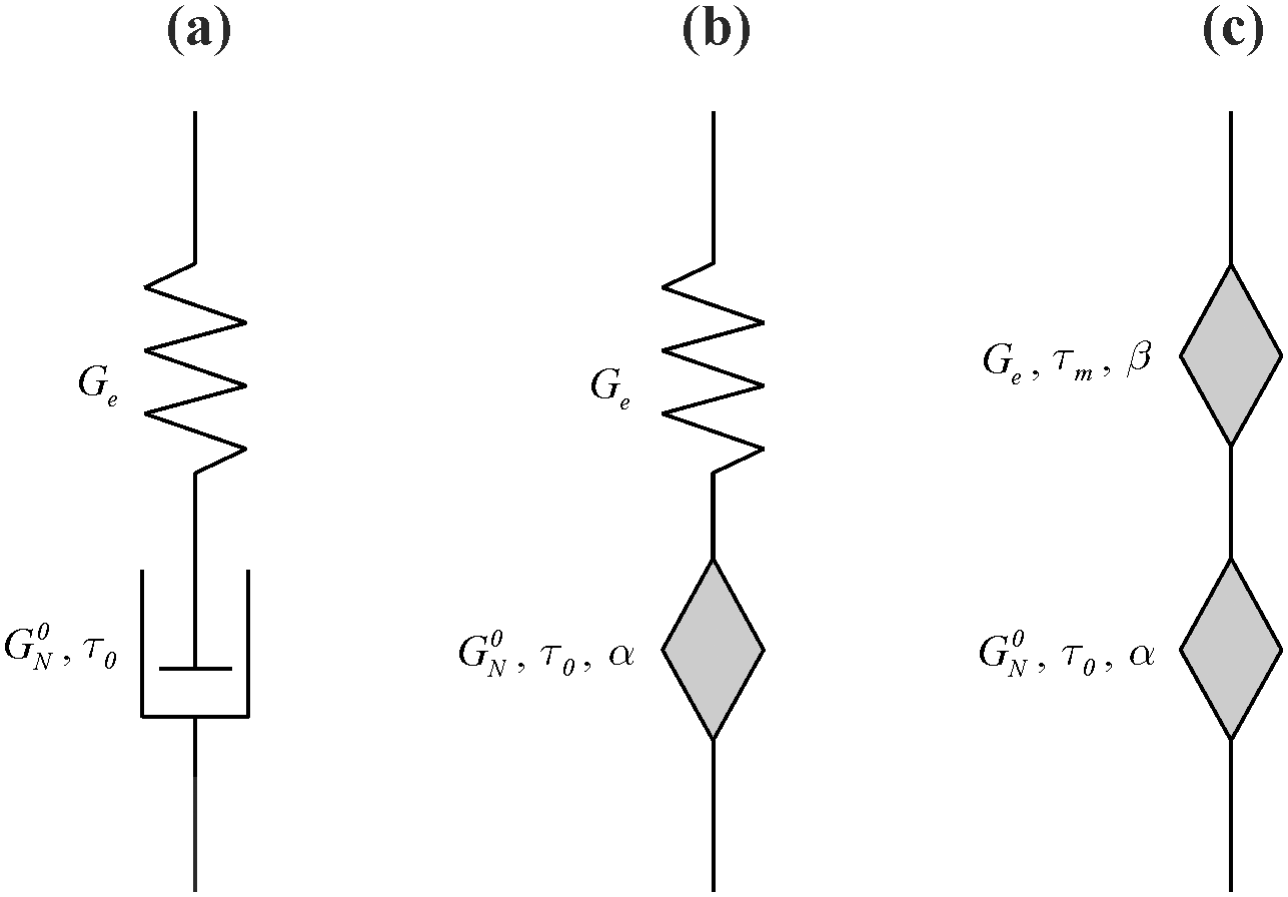
Maxwell-type models. (a) classical; (b) fractional with one springpot; (c) fractional with two springpots.

The classical Maxwell model (CMM) is composed of serially connected Hooke and Newton elements (Fig 2a). The total shear stress is equal to shear stresses acting on both elements Eq (1) and the total shear strain is the sum of the deformations of spring and dashpot Eq (2) [3,10].

$$\sigma_{tot}=\sigma_{s}=\sigma_{d}$$ (1)

$$\gamma_{tot}=\gamma_{s}+\gamma_{d}$$ (2)

where *σ*_*tot*_ is the total shear stress; *σ*_*s*_ is the shear stress acting on spring, expressed by Eq (3); *σ*_*d*_ is the shear stress acting on dashpot, expressed by Eq (4); *γ*_*tot*_ is the total shear strain; *γ*_*s*_ is the shear strain of spring; and *γ*_*d*_ is the shear strain of dashpot.

$$\sigma \left(t\right)=G_{e}\gamma \left(t\right)$$ (3)

$$\sigma \left(t\right)=\eta \frac{\text{d}\gamma \left(t\right)}{\text{d}t}=G_{N}^{0}\tau_{0}\frac{\text{d}\gamma \left(t\right)}{\text{d}t}$$ (4)

where *σ(t)* is the shear stress; *γ(t)* is the shear strain;*η* is the viscosity; *τ*_*0*_ is the characteristic relaxation time; *G*_*e*_ is the equilibrium modulus; $G_{N}^{0}$ is the plateau modulus; and *t* is time.

By differentiating Eq (2) and substituting Eqs (3) and (4), the constitutive equation of the model (5) is obtained. The stress-strain relation contains a single relaxation time *τ*_*0*_ and two different elastic moduli *G*_*e*_ and $G_{N}^{0}$:

$$\sigma \left(t\right)+\frac{G_{N}^{0}\tau_{0}}{G_{e}}\frac{\text{d}\sigma \left(t\right)}{\text{d}t}=G_{N}^{0}\tau_{0}\frac{\text{d}\gamma \left(t\right)}{\text{d}t}$$ (5)

Carrying out Fourier transform of Eq (5) according to the Eqs (6) and (7)

$$z\cdot f\left(t\right)\overset{\text{F}}{\to }z\cdot \hat{f}\left(\omega \right)$$ (6)

$$\frac{{\text{d}}^{m}f\left(t\right)}{\text{d}t^{m}}\overset{\text{F}}{\to }{\left(i\omega \right)}^{m}\hat{f}\left(\omega \right)$$ (7)

and knowing that the ratio of obtained stress and strain transforms defines complex modulus G* Eq (8) [24], the equation describing the value of complex modulus G* as a function of oscillation frequency *ω* for the classical Maxwell model (CMM) is obtained Eq (9).

$$G*=\frac{\hat{\sigma }\left(\omega \right)}{\hat{\gamma }\left(\omega \right)}$$ (8)

$$G*\left(\omega \right)=\frac{iG_{e}G_{N}^{0}\tau_{0}\omega }{G_{e}+iG_{N}^{0}\tau_{0}\omega }$$ (9)

where *f(t)* is the original function; $\hat{f}\left(\omega \right)$ is the Fourier transform of the function *f(t)*; *i* is the imaginary unit; *m* is the order of derivative; and *z* is the constant.

Separating real and imaginary parts of Eq (9), the equations describing storage modulus *G’* Eq (10) and loss modulus *G”* Eq (11), respectively, are obtained.

$$G\prime \left(\omega \right)=\frac{G_{e}{\left(G_{N}^{0}\tau_{0}\omega \right)}^{2}}{G_{e}{}^{2}+{\left(G_{N}^{0}\tau_{0}\omega \right)}^{2}}$$ (10)

$$G\prime \prime \left(\omega \right)=\frac{G_{e}{}^{2}G_{N}^{0}\tau_{0}\omega }{G_{e}{}^{2}+{\left(G_{N}^{0}\tau_{0}\omega \right)}^{2}}$$ (11)

**Fig 3:**
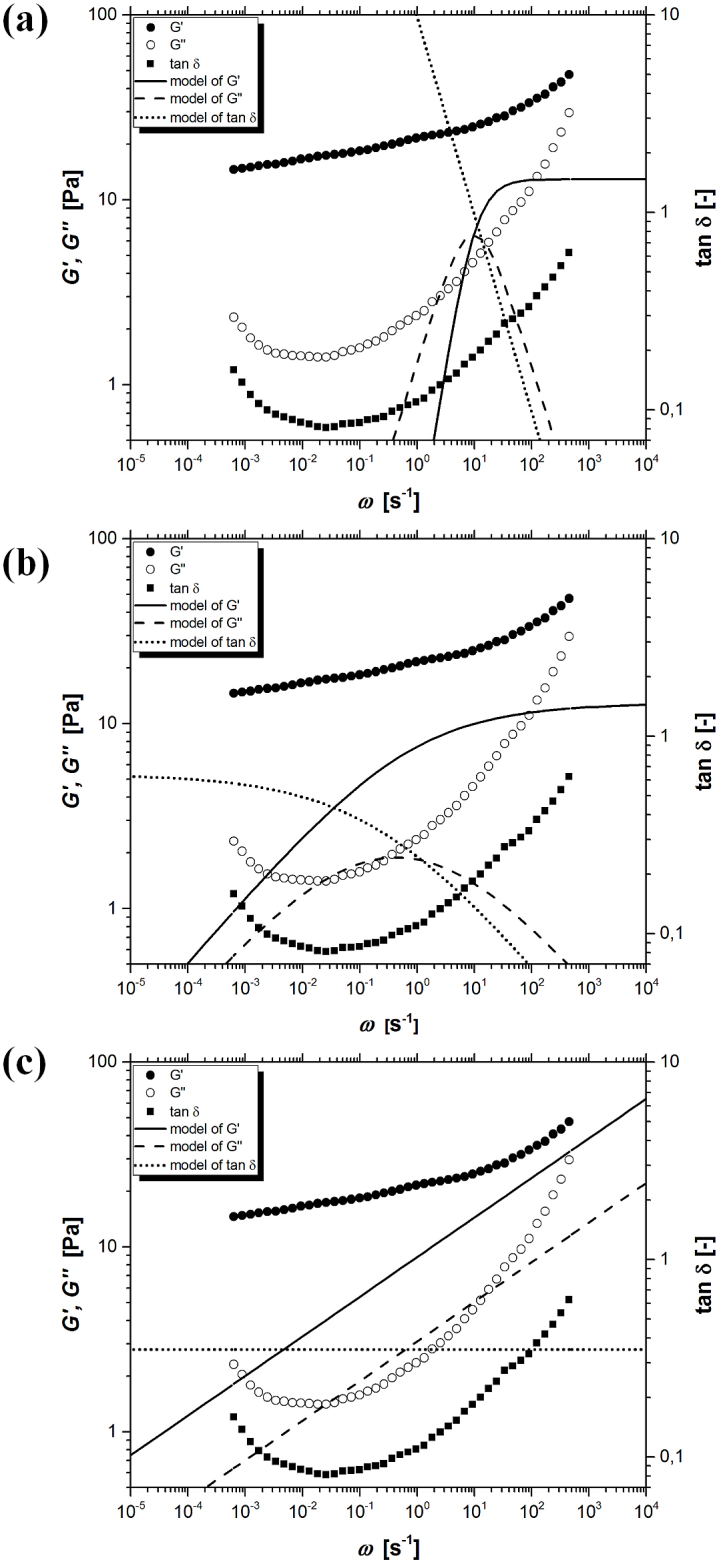
The experimental and model values of storage modulus *G’*, loss modulus *G”* and tangent of loss angle *δ* as a function of oscillation frequency *ω*, for kuzu starch pastes when temperature and time of pasting were 90°C and 30 min, respectively. (a) the classical Maxwell model (CMM), (b) the fractional Maxwell model with one springpot (FMM1), (c) the fractional Maxwell model with two springpots (FMM2).

Fig 3a shows the experimental values of storage modulus *G’*, loss modulus *G”*, tangent of loss angle *δ* and model curves resulting from the classical Maxwell model (CMM) for kuzu starch pastes which were pasted at 90°C for 30 min. Presented model contains only three rheological parameters (*τ*_*0*_, *G*_*e*_, $G_{N}^{0}$) and is completely inadequate to describe the obtained experimental data. The simplest classical form of Maxwell model provides a completely different trend for modeling curves and experimental points.

In order to improve the description of the experimental data, it was proposed to introduce the fractional Maxwell model with one built-in springpot (FMM1). In this model, the dashpot has been replaced with a Scott-Blair element (Fig 2b). The behavior of the springpot-type element is expressed by Eq (12) [13,14]:

$$\sigma \left(t\right)=G_{N}^{0}\tau_{0}{}^{\alpha }\frac{d^{\alpha }\gamma (t)}{dt^{\alpha }}$$ (12)

where *α* is the fractional exponent.

By differentiating Eq (2) and substituting Eqs (3) and (12), the constitutive equation of the model (13) is obtained. The stress-strain relation contains a single relaxation time *τ*_*0*_, two different elastic moduli *G*_*e*_,$G_{N}^{0}$, and fractional exponent *α*:

$$\sigma \left(t\right)+\frac{G_{N}^{0}\tau_{0}{}^{\alpha }}{G_{e}}\frac{{\text{d}}^{\alpha }\sigma \left(t\right)}{\text{d}t^{\alpha }}=G_{N}^{0}\tau_{0}{}^{\alpha }\frac{{\text{d}}^{\alpha }\gamma \left(t\right)}{\text{d}t^{\alpha }}$$ (13)

Using Eqs (6), (7) and (8), the equation describing complex modulus G* as a function of oscillation frequency *ω* for the fractional Maxwell model with one springpot (FMM1) is obtained:

$$G*\left(\omega \right)=\frac{G_{e}G_{N}^{0}{\left(i\tau_{0}\omega \right)}^{\alpha }}{G_{e}+G_{N}^{0}{\left(i\tau_{0}\omega \right)}^{\alpha }}$$ (14)

Separating real and imaginary parts of Eq (14)—with the use of Eq (15) [21]–the equations describing storage modulus *G’* Eq (16) and loss modulus *G”* Eq (17), respectively, are obtained.

$$i^{n}=\text{cos}\left(n\frac{\pi }{2}\right)+i\cdot \text{sin}\left(n\frac{\pi }{2}\right)$$ (15)

$$G\prime \left(\omega \right)=\frac{G_{e}{}^{2}G_{N}^{0}{\left(\tau_{0}\omega \right)}^{\alpha }\text{cos}\left(\alpha \frac{\pi }{2}\right)+G_{e}{\left(G_{N}^{0}\right)}^{2}{\left(\tau_{0}\omega \right)}^{2\alpha }}{G_{e}{}^{2}+2G_{e}G_{N}^{0}{\left(\tau_{0}\omega \right)}^{\alpha }\text{cos}\left(\alpha \frac{\pi }{2}\right)+{\left(G_{N}^{0}\right)}^{2}{\left(\tau_{0}\omega \right)}^{2\alpha }}$$ (16)

$$G\prime \prime \left(\omega \right)=\frac{G_{e}{}^{2}G_{N}^{0}{\left(\tau_{0}\omega \right)}^{\alpha }\text{sin}\left(\alpha \frac{\pi }{2}\right)}{G_{e}{}^{2}+2G_{e}G_{N}^{0}{\left(\tau_{0}\omega \right)}^{\alpha }\text{cos}\left(\alpha \frac{\pi }{2}\right)+{\left(G_{N}^{0}\right)}^{2}{\left(\tau_{0}\omega \right)}^{2\alpha }}$$ (17)

where *n* is the exponent of imaginary unit.

Fig 3b shows the experimental values of storage modulus *G’*, loss modulus *G”*, tangent of loss angle *δ* and model curves resulting from the fractional Maxwell model with one springpot (FMM1). Presented four-parameter model (*τ*_*0*_, *G*_*e*_, $G_{N}^{0}$, *α*) describes the experimental data with an error smaller by several magnitude orders in comparison to the classical Maxwell model (CMM). However, accurate description of the experimental data is still unsatisfactory.

Further expansion of the model by replacing the elastic Hook component with a Scott-Blair element led to the creation of the fractional Maxwell model with two built-in springpots (FMM2) (Fig 2c). Additional springpot is associated with equilibrium modulus *G*_*e*_, characteristic (the longest) relaxation time *τ*_*m*_ and fractional exponent *β* by the following relation:

$$\sigma \left(t\right)=G_{e}\tau_{m}{}^{\beta }\frac{d^{\beta }\gamma (t)}{dt^{\beta }}$$ (18)

By differentiating Eq (2) according to the rule Eq (19) [10] and substituting Eqs (12) and (18), the constitutive equation of the model (20) is obtained. The stress-strain relation contains two relaxation times *τ*_*0*_, *τ*_*m*_, two different elastic moduli *G*_*e*_,$G_{N}^{0}$ and two fractional exponents *α*, *β*.

$$\frac{{\text{d}}^{\alpha }}{\text{d}t^{\alpha }}\frac{{\text{d}}^{\beta }}{\text{d}t^{\beta }}=\frac{{\text{d}}^{\alpha +\beta }}{\text{d}t^{\alpha +\beta }}$$ (19)

$$\sigma \left(t\right)+\frac{G_{N}^{0}\tau_{0}{}^{\alpha }}{G_{e}\tau_{m}{}^{\beta }}\frac{{\text{d}}^{\alpha -\beta }\sigma \left(t\right)}{\text{d}t^{\alpha -\beta }}=G_{N}^{0}\tau_{0}{}^{\alpha }\frac{{\text{d}}^{\alpha }\gamma \left(t\right)}{\text{d}t^{\alpha }}$$ (20)

Using Eqs (6), (7) and (8), the equation describing complex modulus G* as a function of oscillation frequency *ω* for the fractional Maxwell model with two springpots (FMM2) is obtained:

$$G*\left(\omega \right)=\frac{G_{e}G_{N}^{0}{\left(i\tau_{0}\omega \right)}^{\alpha }{\left(i\tau_{m}\omega \right)}^{\beta }}{G_{N}^{0}{\left(i\tau_{0}\omega \right)}^{\alpha }+G_{e}{\left(i\tau_{m}\omega \right)}^{\beta }}$$ (21)

Separating real and imaginary parts of Eq (21)—with the use of Eq (15)—the equations describing storage modulus *G’* Eq (22) and loss modulus *G”* Eq (23), respectively, are obtained.

$$G\prime \left(\omega \right)=\frac{G_{e}G_{N}^{0}{\left(\tau_{0}\omega \right)}^{\alpha }{\left(\tau_{m}\omega \right)}^{\beta }\left[A\text{cos}\left(\left(\alpha +\beta \right)\frac{\pi }{2}\right)+B\text{sin}\left(\left(\alpha +\beta \right)\frac{\pi }{2}\right)\right]}{A^{2}+B^{2}}$$ (22)

$$G\prime \prime \left(\omega \right)=\frac{G_{e}G_{N}^{0}{\left(\tau_{0}\omega \right)}^{\alpha }{\left(\tau_{m}\omega \right)}^{\beta }\left[A\text{sin}\left(\left(\alpha +\beta \right)\frac{\pi }{2}\right)-B\text{cos}\left(\left(\alpha +\beta \right)\frac{\pi }{2}\right)\right]}{A^{2}+B^{2}}$$ (23)

where:

$$A=G_{N}^{0}{\left(\tau_{0}\omega \right)}^{\alpha }\text{cos}\left(\alpha \frac{\pi }{2}\right)+G_{e}{\left(\tau_{m}\omega \right)}^{\beta }\text{cos}\left(\beta \frac{\pi }{2}\right)$$ (24)

$$B=G_{N}^{0}{\left(\tau_{0}\omega \right)}^{\alpha }\text{sin}\left(\alpha \frac{\pi }{2}\right)+G_{e}{\left(\tau_{m}\omega \right)}^{\beta }\text{sin}\left(\beta \frac{\pi }{2}\right)$$ (25)

The fractional Maxwell model with two springpot-type elements (FMM2) contains six parameters (*τ*_*0*_, *τ*_*m*_, *G*_*e*_, $G_{N}^{0}$, *α*, *β*) which leads to a further increase in the quality of model fitting (Fig 3c). In the case of this model, relatively good description of loss modulus *G”* in the range of intermediate oscillation frequencies *ω* was achieved. The improvement in the description of storage modulus *G’* for large values of the oscillation frequencies *ω* was also obtained. On the other hand, the trend of experimental data and model predictions concerning the tangent of loss angle *δ* are fundamentally different.

### Kelvin-Voigt-type models

**Fig 4:**
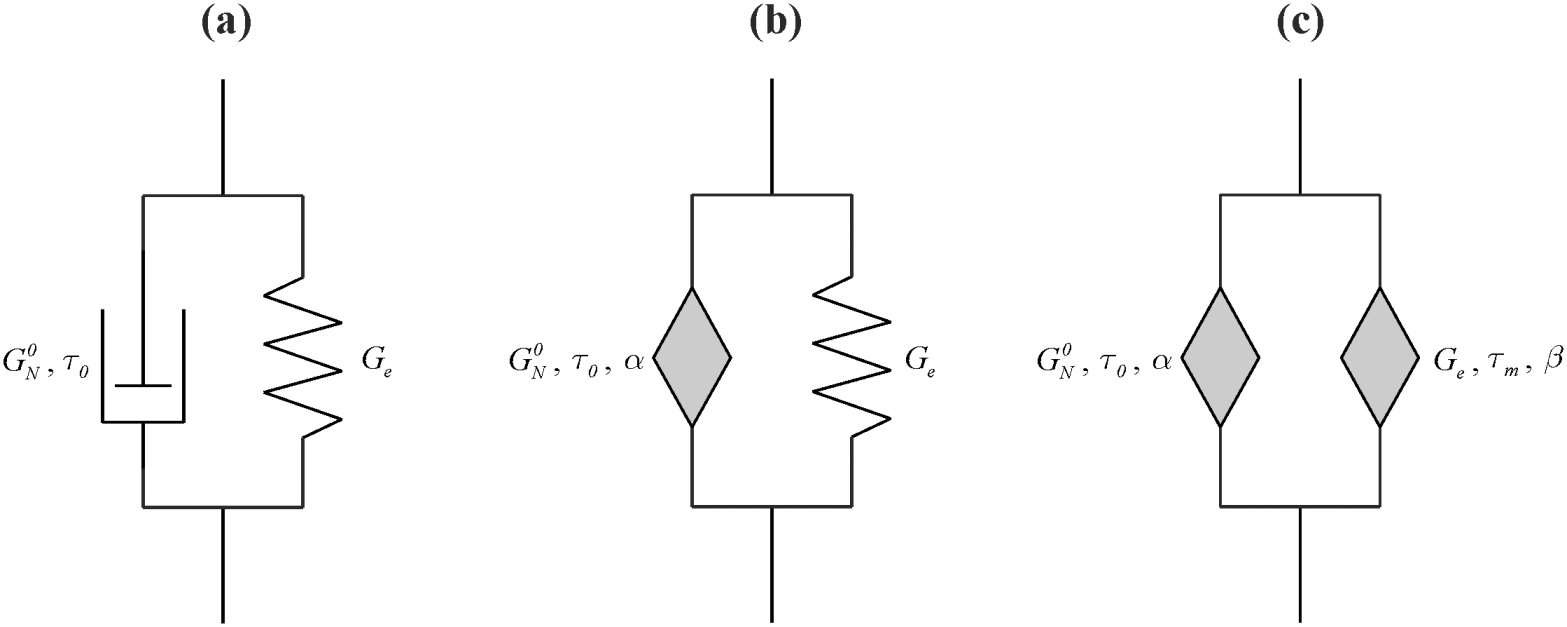
Kelvin-Voigt-type models. (a) classical; (b) fractional with one springpot; (c) fractional with two springpots.

The classical Kelvin-Voigt model (CKVM) is composed of parallel-connected Hooke and Newton elements (Fig 4a). In this case, the total shear stress is the sum of shear stresses acting on both elements Eq (26), and the shear strain is the same for spring and dashpot Eq (27) [3,10].

$$\sigma_{tot}=\sigma_{s}+\sigma_{d}$$ (26)

$$\gamma_{tot}=\gamma_{s}=\gamma_{d}$$ (27)

Substituting Eqs (3) and (4) into Eq (26), the constitutive equation of the model (28) is obtained. The stress-strain relation contains a single relaxation time *τ*_*0*_ and two different elastic moduli *G*_*e*_,$G_{N}^{0}$:

$$\sigma \left(t\right)=G_{e}\gamma \left(t\right)+G_{N}^{0}\tau_{0}\frac{\text{d}\gamma \left(t\right)}{\text{d}t}$$ (28)

Using Eqs (6), (7) and (8), the equation describing complex modulus G* as a function of oscillation frequency *ω* for the classical Kelvin-Voigt model (CKVM) is obtained:

$$G*\left(\omega \right)=G_{e}+iG_{N}^{0}\tau_{0}\omega$$ (29)

Separating real and imaginary parts of Eq (29), the equations describing storage modulus *G’* Eq (30) and loss modulus *G”* Eq (31), respectively, are obtained.

$$G\prime \left(\omega \right)=G_{e}$$ (30)

$$G\prime \prime \left(\omega \right)=G_{N}^{0}\tau_{0}\omega$$ (31)

**Fig 5:**
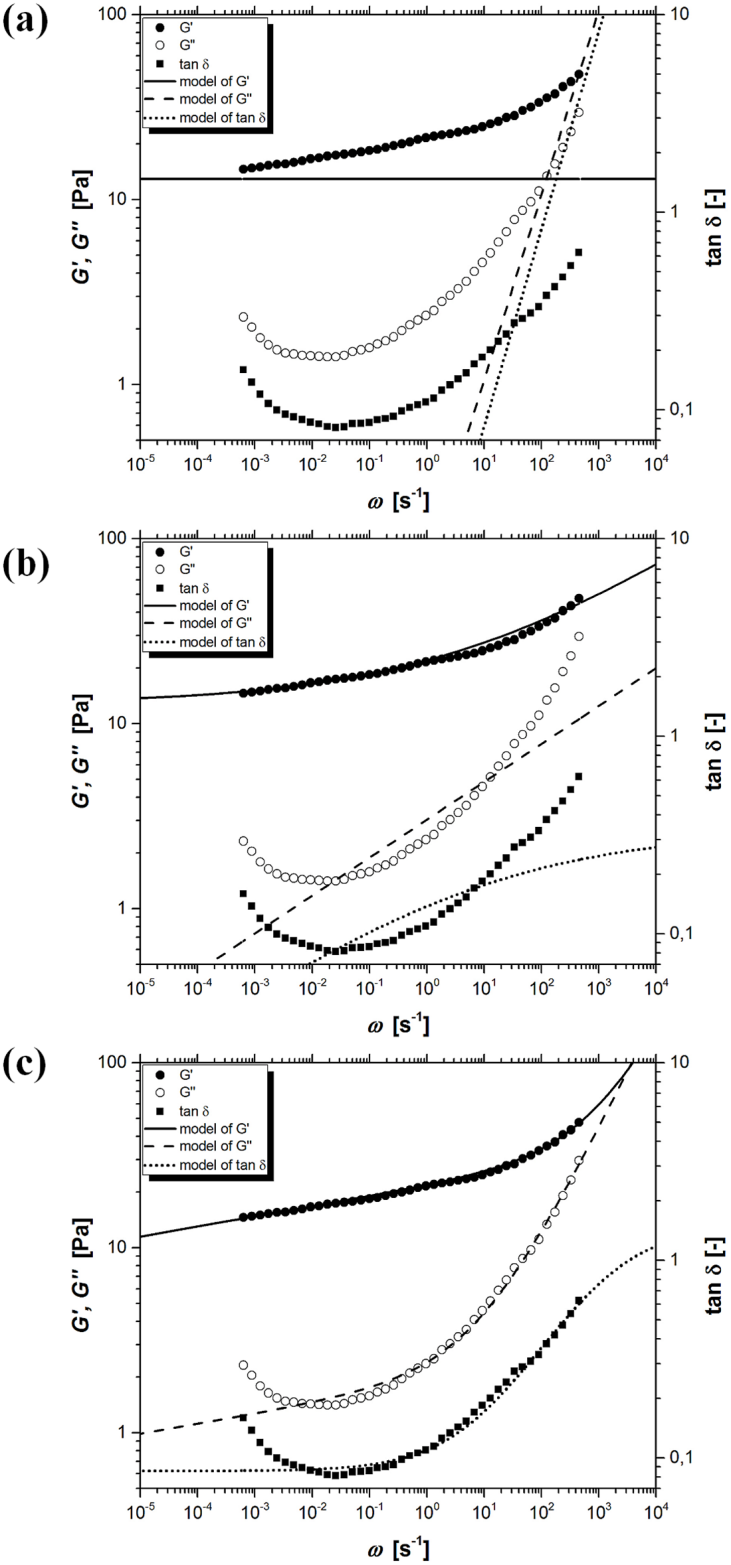
The experimental and model values of storage modulus *G’*, loss modulus *G”* and tangent of loss angle *δ* as a function of oscillation frequency *ω*, for kuzu starch pastes when temperature and time of pasting were 90°C and 30 min, respectively. (a) the classical Kelvin-Voigt model (CKVM), (b) the fractional Kelvin-Voigt model with one springpot (FKVM1), (c) the fractional Kelvin-Voigt model with two springpots (FKVM2).

Fig 5a shows the experimental values of storage modulus *G’*, loss modulus *G”*, tangent of loss angle *δ* and model curves resulting from the classical Kelvin-Voigt model (CKVM). This model—Eqs (30) and (31)–describes the experimental data with a much smaller error than the corresponding classical Maxwell model (CMM), but still it cannot be used because the trends of model curves and experimental points are fundamentally different.

Therefore, there was an attempt to modify the classical Kelvin-Voigt model (CKVM) by replacing the dashpot with a Scott-Blair element (Fig 4b). Substituting Eqs (3) and (12) into Eq (26), the constitutive equation of the fractional Kelvin-Voigt model with one built-in springpot (FKVM1) Eq (32) is obtained. The stress-strain relation contains a single relaxation time *τ*_*0*_, two different elastic moduli *G*_*e*_,$G_{N}^{0}$ and fractional exponent *α*:

$$\sigma \left(t\right)=G_{e}\gamma \left(t\right)+G_{N}^{0}\tau_{0}{}^{\alpha }\frac{{\text{d}}^{\alpha }\gamma \left(t\right)}{\text{d}t^{\alpha }}$$ (32)

Using Eqs (6), (7) and (8), the equation describing complex modulus G* as a function of oscillation frequency *ω* for the fractional Kelvin-Voigt model with one springpot (FKVM1) is obtained:

$$G*\left(\omega \right)=G_{e}+G_{N}^{0}{\left(i\tau_{0}\omega \right)}^{\alpha }$$ (33)

Separating real and imaginary parts of Eq (33)—with the use of Eq (15)—the equations describing storage modulus *G’* Eq (34) and loss modulus *G”* Eq (35), respectively, are obtained.

$$G\prime \left(\omega \right)=G_{e}+G_{N}^{0}{\left(\tau_{0}\omega \right)}^{\alpha }\text{cos}\left(\alpha \frac{\pi }{2}\right)$$ (34)

$$G\prime \prime \left(\omega \right)=G_{N}^{0}{\left(\tau_{0}\omega \right)}^{\alpha }\text{sin}\left(\alpha \frac{\pi }{2}\right)$$ (35)

Replacement of the dashpot with a Scott-Blair element significantly improves a description of the experimental data by means of the fractional Kelvin-Voigt model with one springpot (FKVM1) (Fig 5b). Presented four-parameter fractional model (*τ*_*0*_, *G*_*e*_, $G_{N}^{0}$, *α*) very well captures the course of storage modulus *G’* in the whole investigated range of oscillation frequencies *ω*. While in case of loss modulus *G”* and tangent of loss angle *δ* a substantial improvement in description of the experimental data in the range of intermediate values of oscillation frequencies *ω*, was obtained.

Further modification of the Kelvin-Voigt model by replacing the elastic Hooke element with another Scott-Blair component allowed to obtain the fractional Kelvin-Voigt model with two built-in springpots (FKVM2) (Fig 4c). Substituting Eqs (12) and (18) into Eq (26), the constitutive equation of the model Eq (36) is obtained. The stress-strain relation contains two relaxation times *τ*_*0*_, *τ*_*m*_, two different elastic moduli *G*_*e*_,$G_{N}^{0}$ and two fractional exponents *α*, *β*:

$$\sigma \left(t\right)=G_{N}^{0}\tau_{0}{}^{\alpha }\frac{{\text{d}}^{\alpha }\gamma \left(t\right)}{\text{d}t^{\alpha }}+G_{e}\tau_{m}{}^{\beta }\frac{{\text{d}}^{\beta }\gamma \left(t\right)}{\text{d}t^{\beta }}$$ (36)

Using Eqs (6), (7) and (8), the equation describing complex modulus G* as a function of oscillation frequency *ω* for the fractional Kelvin-Voigt model with two springpots (FKVM2) is obtained:

$$G\;*\left(\omega \right)=G_{N}^{0}{\left(i\tau_{0}\omega \right)}^{\alpha }+G_{e}{\left(i\tau_{m}\omega \right)}^{\beta }$$ (37)

Separating real and imaginary parts of Eq (37)—with the use of Eq (15)—the equations describing storage modulus *G’* Eq (38) and loss modulus *G”* Eq (39), respectively, are obtained.

$$G\prime \left(\omega \right)=G_{N}^{0}{\left(\tau_{0}\omega \right)}^{\alpha }\text{cos}\left(\alpha \frac{\pi }{2}\right)+G_{e}{\left(\tau_{m}\omega \right)}^{\beta }\text{cos}\left(\beta \frac{\pi }{2}\right)$$ (38)

$$G\prime \prime \left(\omega \right)=G_{N}^{0}{\left(\tau_{0}\omega \right)}^{\alpha }\text{sin}\left(\alpha \frac{\pi }{2}\right)+G_{e}{\left(\tau_{m}\omega \right)}^{\beta }\text{sin}\left(\beta \frac{\pi }{2}\right)$$ (39)

Presented six-parameter fractional Kelvin-Voigt model (*τ*_*0*_, *τ*_*m*_, *G*_*e*_, $G_{N}^{0}$, *α*, *β*) with two springpot-type elements (FKVM2) very well describes the course of storage modulus *G’* over the entire range of oscillation frequencies *ω* (Fig 5c). Trends in loss modulus *G”* and tangent of loss angle *δ* are also very well described by the model curves, except for a narrow range of the smallest oscillation frequencies *ω*.

The above applicability analysis of various forms of Maxwell and Kelvin-Voigt models indicates that it is necessary to propose modifications to these models that allow for a description of the experimental data on viscoelastic solids in broad range of oscillation frequencies *ω*.Modification of fractional rheological models

The two-element rheological models are not able to describe the dynamic behavior of real materials in a wide range of oscillation frequencies *ω* with an acceptable accuracy [9]. In order to improve the description quality of the loss peak on loss modulus curve *G”* (Figs 3 and 5), the paper proposes a modification of the fractional Maxwell and Kelvin-Voigt models with two built-in springpots (FMM2 and FKVM2) by adding to the Eqs (23) and (39) a new component called the network durability. Rayleigh dimensional analysis indicates that a function describing the dependence of loss modulus *G”* from the rest of parameters associated with the phenomenon of energy dissipation must fulfill the following condition:

$$f\left(G\prime \prime ,\omega ,G_{N}^{0},G_{e},\eta_{0}\right)=0$$ (40)

where *η*_*0*_ is the Newtonian steady state shear viscosity.

According to the Fourier principle, all the laws of physics are expressed by dimensionally homogeneous equations [25]. This makes it possible to write Eq (41), which after substituting the appropriate units for all variables, takes the form Eq (42).

$${\left(G\prime \prime \right)}^{-1}\cdot {\left(\omega \right)}^{a}\cdot {\left(G_{N}^{0}\right)}^{b}\cdot {\left(G_{e}\right)}^{c}\cdot {\left(\eta_{0}\right)}^{d}=1$$ (41)

$${\left(Pa\right)}^{-1}\cdot {\left(s^{-1}\right)}^{a}\cdot {\left(Pa\right)}^{b}\cdot {\left(Pa\right)}^{c}\cdot {\left(Pa\cdot s\right)}^{d}=1$$ (42)

The dimensional compatibility on both sides of the Eq (42) occurs when:

−1+b+c+d=0     ∧    −a+d=0 (43)

Presented problem has three possible solutions under the assumption that the exponent of oscillation frequency *ω* takes the value of *a* = −1 and the remaining exponents are natural numbers:

b=2  ;  c=0     ∨    b=1  ;  c=1     ∨    b=0  ;  c=2  (44)

The analysis of experimental data has allowed to determine that the best fit to the loss peak on *G”* curve is achieved when *b* = 2 and *c* = 0.

The network durability *D* thus obtained characterizes flowing abilities of the imaginary elementary cells of biopolymer network—units locked by a minimal number of nodes and having the individual movement ability:

$$D=\frac{{\left(G_{N}^{0}\right)}^{2}}{\eta_{0}\cdot \;\omega }$$ (45)

Substituting Eq (45) into Eqs (23) and (39), the equations describing the value of loss modulus *G”* as a function of oscillation frequency *ω* for the modified fractional Maxwell model with two springpots (MFMM2) Eq (46) and the modified fractional Kelvin-Voigt model with two springpots (MFKVM2) Eq (47), respectively, are obtained. The proposed modified fractional models increase the ability to describe both fast and slow processes of energy dissipation.

$$G\prime \prime \left(\omega \right)=\frac{G_{e}G_{N}^{0}{\left(\tau_{0}\omega \right)}^{\alpha }{\left(\tau_{m}\omega \right)}^{\beta }\left[A\text{sin}\left(\left(\alpha +\beta \right)\frac{\pi }{2}\right)-B\text{cos}\left(\left(\alpha +\beta \right)\frac{\pi }{2}\right)\right]}{A^{2}+B^{2}}+\frac{{\left(G_{N}^{0}\right)}^{2}}{\eta_{0}\cdot \;\omega }$$ (46)

$$G\prime \prime \left(\omega \right)=G_{N}^{0}{\left(\tau_{0}\omega \right)}^{\alpha }\text{sin}\left(\alpha \frac{\pi }{2}\right)+G_{e}{\left(\tau_{m}\omega \right)}^{\beta }\text{sin}\left(\beta \frac{\pi }{2}\right)+\frac{{\left(G_{N}^{0}\right)}^{2}}{\eta_{0}\cdot \omega }$$ (47)

**Fig 6:**
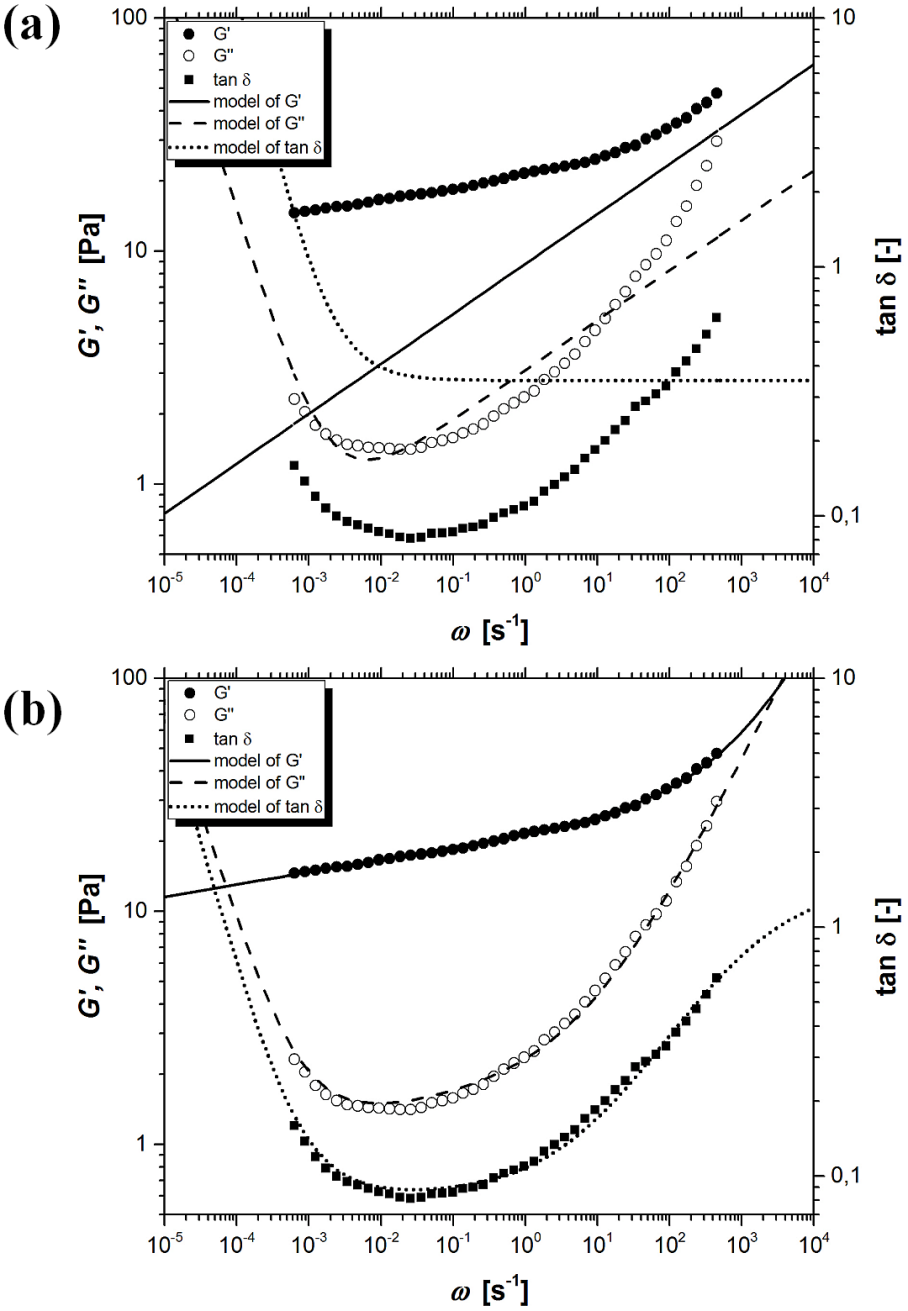
The experimental and model values of storage modulus *G’*, loss modulus *G”* and tangent of loss angle *δ* as a function of oscillation frequency *ω*, for kuzu starch pastes when temperature and time of pasting were 90°C and 30 min, respectively. (a) the modified fractional Maxwell model with two springpots (MFMM2), (b) the modified fractional Kelvin-Voigt model with two springpots (MFKVM2).

Fig 6 shows the experimental values of storage modulus *G’*, loss modulus *G”*, tangent of loss angle *δ* and model curves resulting from the seven-parameter (*τ*_*0*_, *τ*_*m*_, *G*_*e*_, $G_{N}^{0}$, *α*, *β*, *η*_*0*_) modified fractional models with two built-in springpots: Maxwell-type (MFMM2) (Fig 6a) and Kelvin-Voigt-type (MFKVM2) (Fig 6b). In the case of MFKVM2, a modification in the form of additional component called the network durability *D* allows for a significant improvement in a description of the experimental data over the entire range of oscillation frequencies *ω*.

### Parameters of fractional rheological models and their limitations

Presented fractional rheological models of Maxwell and Kelvin-Voigt—Eqs (10) and (11); (16) and (17); (22) and (23); (30) and (31); (34) and (35); (38) and (39); (22) and (46); (38) and (47)–contain seven rheological parameters (Table 1), which describe a number of viscoelastic properties of a given material:

- plateau modulus $G_{N}^{0}$ –represents the power of biopolymer network and its resistance to aging during time; value of the parameter was determined using the minimum method [26]–it is based on the assumption that the plateau modulus $G_{N}^{0}$ corresponds to the value of storage modulus *G’* at the oscillation frequency *ω* in which the tangent of loss angle *δ* reaches a minimum:

$$G_{N}^{0}=G\prime {\left(\omega \right)}_{\text{tan}\left(\delta \right)\to \text{min}}$$ (48)

- equilibrium modulus *G*_*e*_—illustrates the total elasticity of biopolymer network; value of the parameter was taken as equal to the inverse of the intercept in the equation of line tangent to the shear creep compliance curve *J(t)* at endpoint [16];
- characteristic relaxation times *τ*_*0*_ and *τ*_*m*_—represent the shortest and the longest time required to complete stress relaxation in the biopolymer network; values of the parameters correspond to the inverses of oscillation frequencies *ω* at which the dynamic curves *G’* and *G”* intersect themselves;
- fractional exponents *α* and *β*–indicate which properties dominate in the material: these parameters take values from 0 (for perfectly elastic solid) to 1 (for ideal Newtonian fluid);
- Newtonian steady state shear viscosity *η*_*0*_ —characterizes flowing abilities of the elementary units of biopolymer network; value of the parameter was taken as equal to the inverse of the slope in the equation of line tangent to the shear creep compliance curve *J(t)* at endpoint [16].

**Table 1:** The list of parameters of the proposed rheological models.

	CMM	FMM1	FMM2	MFMM2	CKVM	FKVM1	FKVM2	MFKVM2
**Eqs**	(10), (11)	(16), (17)	(22), (23)	(22), (46)	(30), (31)	(34), (35)	(38), (39)	(38), (47)
***G*** ^***0***^_***N***_	+	+	+	+	+	+	+	+
***G***_***e***_	+	+	+	+	+	+	+	+
***τ***_***0***_	+	+	+	+	+	+	+	+
***τ***_***m***_			+	+			+	+
***α***		+	+	+		+	+	+
***β***			+	+			+	+
***η***_***0***_				+				+

where: *G* ^*0*^_*N*_—the plateau modulus; *G*_*e*_—the equilibrium modulus; *τ*_*0*_ —the shortest relaxation time; *τ*_*m*_—the longest relaxation time; *α*, *β*–the fractional exponents; *η*_*0*_—the Newtonian steady state shear viscosity; CMM—the classical Maxwell model; FMM1—the fractional Maxwell model with one springpot; FMM2—the fractional Maxwell model with two springpots; MFMM2—the modified fractional Maxwell model with two springpots; CKVM—the classical Kelvin-Voigt model; FKVM1—the fractional Kelvin-Voigt model with one springpot, FKVM2—the fractional Kelvin-Voigt model with two springpots, MFKVM2—the modified fractional Kelvin-Voigt model with two springpots; the sign of "+" means the presence of given parameter in the model.

Thanks to the correlations available in the literature [16,18,22,26–31], it is possible to determine further indicators characterizing the rheological properties of biopolymer, such as:

- plateau compliance $J_{N}^{0}$– representing the force with which the entanglements of the biopolymer network suppress any kind of long-range configurational rearrangements:

$$J_{N}^{0}=\frac{1}{G_{N}^{0}}\;$$ (49)

- steady state compliance *J*_*e*_—being the measure of stored energy in the steady-state flow under the influence of low stresses:

$$J_{e}=\frac{1}{G_{e}}$$ (50)

- dispersion modulus *f*–characterizing the molecular weight distributions of the biopolymer:

$$f=\frac{G_{N}^{0}}{G_{e}}$$ (51)

- coefficient of the network vibration damping *k*–representing the resistance of biopolymer network to oscillatory deformations:

$$k=\frac{G_{N}^{0}-G_{e}}{G_{e}}$$ (52)

- width of the viscoelastic plateau *L*–specifying the polydispersity index of the biopolymer:

$$L=\frac{\tau_{m}}{\tau_{0}}$$ (53)

- cross-linking density *ω*_*0*_—characterizing the structure of biopolymer network:

$$\omega_{0}=\frac{1}{\tau_{0}}$$ (54)

- gel stiffness *S*–representing the degree of fragility, brittleness of gel:

$$S=G_{N}^{0}\tau_{0}{}^{\frac{\alpha +\beta }{2}}$$ (55)

- average entanglement molecular weight *M*_*e*_—being the average molecular weight between topological constraints:

$$M_{e}=\frac{\rho RT}{G_{N}^{0}}$$ (56)

- average molecular weight between cross-links *M*_*c*_—being the average molecular weight of biopolymer chains between two consecutive junctions:

$$M_{c}=\frac{\rho RT}{G_{e}}$$ (57)

where *ρ* is density of biopolymer; *R* is universal gas constant; and *T* is temperature.

According to the second law of thermodynamics, the dynamically deformable real solids must be characterized by positive values of energy dissipation and internal work [32]. It means that the proposed rheological models have physical meaning only when model values of storage modulus *G’* and loss modulus *G”* are positive in the entire analyzed range of oscillation frequencies *ω* [33]. This condition is fulfilled when:

$$0\leq G_{e}\leq \;G_{N}^{0}\;\wedge \;0\leq \tau_{0}\leq \tau_{m}\;\wedge \;0\leq \beta \leq \alpha \leq 1\;\wedge \;0\leq \eta_{0}$$ (58)

### Statistical evaluation of rheological models

In order to determine the quality of the experimental data description by means of the proposed Maxwell and Kelvin-Voigt rheological models, the statistical evaluation referred to tangent of loss angle *δ* Eq (59) was performed.

$$\text{tan }\delta =\frac{G\prime \prime \left(\omega \right)}{G\prime \left(\omega \right)}$$ (59)

The analysis was carried out using the statistical indicators, such as [34,35]:

- mean percentage error (MPE):

$$\text{MPE}=\frac{1}{N}\overset{N}{\underset{j=1}{\sum }}\left[\frac{\text{tan }\delta_{\text{exp},\;j}-\text{tan }\delta_{\text{mod},\;j}}{\text{tan }\delta_{\text{exp},\;j}}\right]\cdot 100$$ (60)

- mean bias error (MBE):

$$\text{MBE}=\frac{1}{N}\overset{N}{\underset{j=1}{\sum }}\left(\text{tan }\delta_{\text{mod},\;j}-\text{tan }\delta_{\text{exp},\;j}\right)$$ (61)

- root mean square error (RMSE):

$$\text{RMSE}={\left[\frac{1}{N}\overset{N}{\underset{j=1}{\sum }}{\left(\text{tan }\delta_{\text{exp},\;j}-\text{tan }\delta_{\text{mod},\;j}\right)}^{2}\right]}^{\frac{1}{2}}$$ (62)

- modelling efficiency (EF):

$$\text{EF}=\frac{\overset{N}{\underset{j=1}{\sum }}{\left(\text{tan }\delta_{\text{exp},\;j}-\text{tan }\delta_{\text{exp},\;\mathrm{ave}}\right)}^{2}-\overset{N}{\underset{j=1}{\sum }}{\left(\text{tan }\delta_{\text{mod},\;j}-\text{tan }\delta_{\text{exp},\;j}\right)}^{2}}{\overset{N}{\underset{j=1}{\sum }}{\left(\text{tan }\delta_{\text{exp},\;j}-\text{tan }\delta_{\text{exp},\;\mathrm{ave}}\right)}^{2}}$$ (63)

- chi-square test (χ^2^):

$$\chi^{2}=\frac{\overset{N}{\underset{j=1}{\sum }}{\left(\text{tan }\delta_{\text{exp},\;j}-\text{tan }\delta_{\text{mod},\;j}\right)}^{2}}{N-n}$$ (64)

where tan *δ*_exp,j_ is the experimental value of tangent of loss angle *δ*; tan *δ*_mod,j_ is the model value of tangent of loss angle *δ*; tan *δ*_exp,ave_ is the average experimental value of tangent of loss angle *δ*; *j* is the index of experimental point; *N* is the number of experimental points; *n* is the number of parameters in the model.

## Results and Discussion

In order to analyze the accuracy of the experimental data description by the proposed modified Maxwell and Kelvin-Voigt models, the new rheological studies for kuzu starch pastes were carried out. The obtained results (Fig 6b) revealed that in the oscillation frequency range from 6.3·10^−4^ to 450 s^-1^ the biopolymer was located in a hyperelastic physical state called the viscoelastic plateau region. This area is associated with changes in the position of chain segments of biopolymer (rotary and sliding movement) in an absence of movement of all macroparticles. It may result in significant deformations of the material even in the case of small external stresses [16,22]. In the entire analyzed range of oscillation frequencies *ω*, the storage modulus *G’* (representing elastic properties of biopolymer) was greater than the loss modulus *G”* (characterizing viscous features of medium).

Table 2 lists the goodness-of-fit indicators for presented in the work rheological models. The analysis showed that the optimal model for describing the dynamic behavior of biopolymers, such as kuzu starch pastes, is modified fractional Kelvin-Voigt model with two built-in springpots (MFKVM2). In this case, for pasting time *t* = 15 min, the mean percentage error was equal to MPE = 2.12%, and the modeling efficiency has reached the value of EF = 0.995. It was also confirmed that the rheological models of Maxwell-type do not work in the case of modeling of viscoelastic solids, that is the media in which elastic properties dominate over viscous properties [36].

**Table 2:** The goodness-of-fit indicators for rheological models presented in the work, for kuzu starch pastes when the temperature of pasting was 90°C.

*t* [min]	Indicator	CMM	FMM1	FMM2	MFMM2	CKVM	FKVM1	FKVM2	MFKVM2
**15**	**MPE [%]**	-1.63·10^6^	-1.68·10^2^	-1.21·10^2^	-2.86·10^2^	29.3	21.5	9.34	2.12
**15**	**MBE [–]**	2.40·10^3^	9.86·10^−2^	9.14·10^−2^	0.326	0.138	-7.16·10^−2^	-1.45·10^−2^	-7.40·10^−4^
**15**	**RMSE [–]**	6.37·10^3^	0.301	0.178	0.526	0.650	0.142	3.44·10^−2^	1.13·10^−2^
**15**	**EF [–]**	1.73·10^9^	-2.87	-0.360	-10.8	-17.0	0.144	0.949	0.995
**15**	**χ** ^**2**^ **[–]**	4.36·10^7^	0.100	3.71·10^−2^	0.331	0.455	2.22·10^−2^	1.38·10^−3^	1.54·10^−4^
**30**	**MPE [%]**	-1.07·10^6^	-1.77·10^2^	-1.73·10^2^	-2.45·10^2^	25.0	13.3	3.59	-1.19
**30**	**MBE [–]**	1.29·10^3^	0.111	0.170	0.260	0.141	-4.66·10^−2^	-5.75·10^−3^	1.21·10^−3^
**30**	**RMSE [–]**	3.41·10^3^	0.312	0.216	0.395	0.664	0.103	1.84·10^−2^	1.17·10^−2^
**30**	**EF [–]**	-6.60·10^8^	-4.51	-1.64	-7.83	-24.0	0.402	0.981	0.992
**30**	**χ** ^**2**^ **[–]**	1.25·10^7^	0.108	5.42·10^−2^	0.187	0.474	1.17·10^−2^	3.95·10^−4^	1.65·10^−4^
**45**	**MPE [%]**	-1.33·10^6^	-1.60·10^2^	-63.7	-1.33·10^2^	25.1	20.8	1.77	-2.25
**45**	**MBE [–]**	1.90·10^3^	0.117	3.91·10^−2^	0.137	0.134	-6.44·10^−2^	-3.84·10^−3^	5.64·10^−3^
**45**	**RMSE [–]**	5.05·10^3^	0.324	0.139	0.238	0.638	0.130	2.12·10^−2^	1.06·10^−2^
**45**	**EF [–]**	-1.44·10^9^	-4.93	-8.78·10^−2^	-2.19	-22.0	4.70·10^−2^	0.975	0.994
**45**	**χ** ^**2**^ **[–]**	2.74·10^7^	0.116	2.25·10^−2^	6.79·10^−2^	0.438	1.87·10^−2^	5.23·10^−4^	1.34·10^−4^
**60**	**MPE [%]**	-1.83·10^6^	-1.34·10^2^	-53.6	-71.3	49.7	38.5	14.2	9.17
**60**	**MBE [–]**	2.85·10^3^	0.106	3.53·10^−2^	6.38·10^−2^	2.07·10^−2^	-8.52·10^−2^	-2.11·10^−2^	-1.19·10^−2^
**60**	**RMSE [–]**	7.56·10^3^	0.295	0.125	0.158	0.375	0.129	3.17·10^−2^	1.48·10^−2^
**60**	**EF [–]**	-3.97·10^9^	-5.03	-8.77·10^−2^	-0.725	-8.77	-0.159	0.930	0.985
**60**	**χ** ^**2**^ **[–]**	6.15·10^7^	9.61·10^−2^	1.83·10^−2^	2.98·10^−2^	0.152	1.85·10^−2^	1.17·10^−3^	2.64·10^−4^
**75**	**MPE [%]**	4.48·10^6^	-1.77·10^2^	-1.15·10^2^	-3.19·10^2^	34.9	21.8	1.56	-4.17
**75**	**MBE [–]**	5.40·10^3^	0.102	7.17·10^−2^	0.301	8.76·10^−2^	-5.86·10^−2^	2.61·10^−3^	4.57·10^−3^
**75**	**RMSE [–]**	1.43·10^4^	0.240	0.141	0.445	0.460	0.117	3.51·10^−2^	7.43·10^−3^
**75**	**EF [–]**	1.38·10^10^	-2.90	-0.349	-12.3	-13.3	7.61·10^−2^	0.917	0.996
**75**	**χ** ^**2**^ **[–]**	2.21·10^8^	6.39·10^−2^	2.33·10^−2^	0.237	0.228	1.51·10^−2^	1.44·10^−3^	6.62·10^−5^

where: *t*–time of pasting; MPE—mean percentage error; MBE—mean bias error; RMSE—root mean square error; EF—modelling efficiency; χ^2^—chi-square test; CMM—the classical Maxwell model; FMM1—the fractional Maxwell model with one springpot; FMM2—the fractional Maxwell model with two springpots; MFMM2—the modified fractional Maxwell model with two springpots; CKVM—the classical Kelvin-Voigt model; FKVM1—the fractional Kelvin-Voigt model with one springpot, FKVM2—the fractional Kelvin-Voigt model with two springpots, MFKVM2—the modified fractional Kelvin-Voigt model with two springpots.

The rheological parameters of Japanese kuzu starch pastes determined on the basis of the modified fractional Kelvin-Voigt model with two springpots—Eqs (38) and (47)–provide a lot of additional information about the structure and viscoelastic properties of the biopolymer in comparison to the classical analysis of dynamic curves *G’* and *G”* and shear creep compliance *J(t)*. This is particularly important in the field of materials science to design the utility and functional characteristics of products.

## Conclusions

The analysis of various types of Maxwell and Kelvin-Voigt rheological models for the description of viscoelastic properties of biopolymers indicated that:

- Maxwell-type rheological models do not work in the case of modeling of viscoelastic solids. The optimal model for describing the dynamic behavior of kuzu starch pastes is modified fractional Kelvin-Voigt model with two built-in springpots (MFKVM2). This model can be an important tool for specialists in the field of materials engineering, to design the structure and rheological properties of the media, which have a direct impact on utility and functional characteristics of products.
- The proposed modification of the fractional Kelvin-Voigt model with two springpots (MFKVM2), consists in introducing an additional component called the network durability *D* into the equation for loss modulus *G”*. It allowed for a significant improvement in the quality of experimental data description in the range of the lowest oscillation frequencies *ω*, which correspond to slow dissipative processes (Table 2). The proposed modulus *D* Eq (45) characterizes the impact of cross-linking on the flowing abilities of imaginary cells of the biopolymer network.
